# Publication Trends of Research on Retinoblastoma During 2001–2021: A 20-Year Bibliometric Analysis

**DOI:** 10.3389/fmed.2021.675703

**Published:** 2021-05-21

**Authors:** Xiang Gu, Minyue Xie, Renbing Jia, Shengfang Ge

**Affiliations:** ^1^Department of Ophthalmology, Ninth People's Hospital, Shanghai JiaoTong University School of Medicine, Shanghai, China; ^2^Shanghai Key Laboratory of Orbital Diseases and Ocular Oncology, Shanghai JiaoTong University School of Medicine, Shanghai, China

**Keywords:** retinoblastoma, publication trends, bibliometric analysis, chemotherapy, citations

## Abstract

**Background:** Retinoblastoma is the most common primary intraocular malignancy of childhood. Despite high survival and eye salvage as the result of various types of therapies, retinoblastoma remains a disease that places a considerable burden on developing countries. Our study attempted to analyse the research trends in retinoblastoma research and compare contributions from different countries, institutions, journals, and authors.

**Methods:** We extracted all publications concerning retinoblastoma from 2001 to 2021 from the Web of Science database. Microsoft Excel and VOSviewer were employed to collect publication data, analyse publication trends, and visualize relevant results.

**Results:** A total of 1,675 publications with 30,148 citations were identified. The United States contributed the most publications (643) and citations (16,931 times) with the highest H-index value (67) as of February 4, 2021. China ranked second in the number of publications (259), while ranking fourth in both citations (2,632 times) and the H-index (26) ranked fourth. The *British Journal of Ophthalmology* was the most productive journal concerning retinoblastoma, and Abramson DH had published the most papers in the field. Keywords were categorized into three clusters; tumor-related research, clinical research, and management-related research. The keywords “intravitreal,” “intraarterial,” and “intravenous” appeared the most frequently, with the average appearing year being 2018.1, 2017.7, and 2017.1, respectively. Management-related research has been recognized as a heavily researched topic in the field.

**Conclusion:** We conclude that the United States, China, and India made the most exceptional contributions in the field of retinoblastoma research, while China still has a disparity between the quantity and quality of publications. Management-related research, including intravitreal, intraarterial, and intravenous chemotherapy was considered as a potential focus for future research.

## Introduction

Retinoblastoma, the most common primary intraocular malignancy of childhood, is initiated by mutation of both *RB1* alleles in a single susceptible developing retinal cell, undergoes the limited proliferation of an *RB1*^−/−^ retinal cell to form a non-malignant retinal tumor and consequently experiences uncontrolled proliferation and malignant transformation based on genetic or epigenetic alterations (1).

The incidence of retinoblastoma is constant without race or gender differences at one case every 15,000 to 20,000 live births worldwide (2). Asia and Africa, which experience large populations and high birth rates, bear the greatest burden of retinoblastoma (3). They also carry the highest mortality of 40 to 70%, compared with 3 to 5% in Europe, Canada, and the USA, which contributes to the delay in diagnosis and the lack of access to canonical treatment (4–6).

The diagnosis of retinoblastoma is often based on special signs and clinical examinations. Leukocoria is the most common clinical feature of retinoblastoma, in which the normal pupillary is replaced by a whitish discoloration as a result of abnormal growth and calcification of the developing intraocular tumor, followed by strabismus, owing to loss of central vision from the growing tumor. Advanced retinoblastoma may manifest with iris color change, enlarged cornea, or non-infective orbital inflammation (7). Commonly, it is sufficient to make the diagnosis employing indirect ophthalmoscopy with the pupil pharmacologically dilated. In addition, ocular ultrasonography can be used to detect calcification and magnetic resonance imaging (MRI) can be used to evaluate invasion of the optic nerve and the existence of trilateral retinoblastoma (8). Owing to the risk of metastasis brought by biopsy, diagnosis of retinoblastoma does not depend on histopathologic examination (9). However, histological examination after enucleation is a way of assessing high-risk features and establish pathological staging to assist the management of retinoblastoma (10).

The management of retinoblastoma underwent dramatic evolution in a short period of time. Enucleation was the major treatment for retinoblastoma until the emergence of external beam radiotherapy (EBRT) in the 1950s. However, radiation considerably enhances the risk of developing a second malignancy in survivors of retinoblastoma, consequently leading to its replacement by chemotherapy in the 1990s (11). Chemotherapy was originally administered intravenously with vincristine, etoposide, and carboplatin as the most common agents, and intravenous chemotherapy (IVC) combined with local treatments achieved over 90% tumor control rates in Group A-C retinoblastoma eyes (12). Notably, subsequent targeted chemotherapy, such as intraarterial chemotherapy (IAC) and intravitreal chemotherapy (IVitC), was introduced to diminish the systemic side effects related to IVC and to increase the salvage rate of advanced intraocular retinoblastoma eyes (13, 14). The reported eye salvage rate for Group D retinoblastoma increased from 47 to 85% thanks to the unitality of IAC (15). Various therapies guarantee that the retinoblastoma patients to choose the optimal management according to their conditions. Sound knowledge of the individual conditions and careful monitoring for recurrences are pivotal for the management of retinoblastoma patients.

Bibliometrics is an optimal choice to evaluate particular research trends concerning a certain field over time (16). Bibliometrics plays an important role in analyzing the quantity and quality of publications, including books and journal articles, by employing a literature system and literature metrology (17). Furthermore, the analysis contributes to characterizing and predicting the development in a specific field and comparing contributions among countries, institutions, journals, and authors (18). Remarkably, bibliometrics has been increasingly prevalent based on its importance in governing policy-making, clinical guidelines and research trends (19–21). However, there have so far been few bibliometric studies in the field of ophthalmology, and even fewer in the field of ocular tumors (22).

The study manifests a general analysis of the present state of global retinoblastoma research based on Web of Science (WOS) data. Bibliometrics was applied to uncover the trends in retinoblastoma research and explore its potential hotspots.

## Methods

### Data Sources and Search Strategies

Based on the fact that the Science Citation Index-Expanded (SCI-E) of WOS was considered the optimal database for bibliometric analysis, a literature search was conducted for the years 2001 to 2021 using the WOS database.

**Figure 1:**
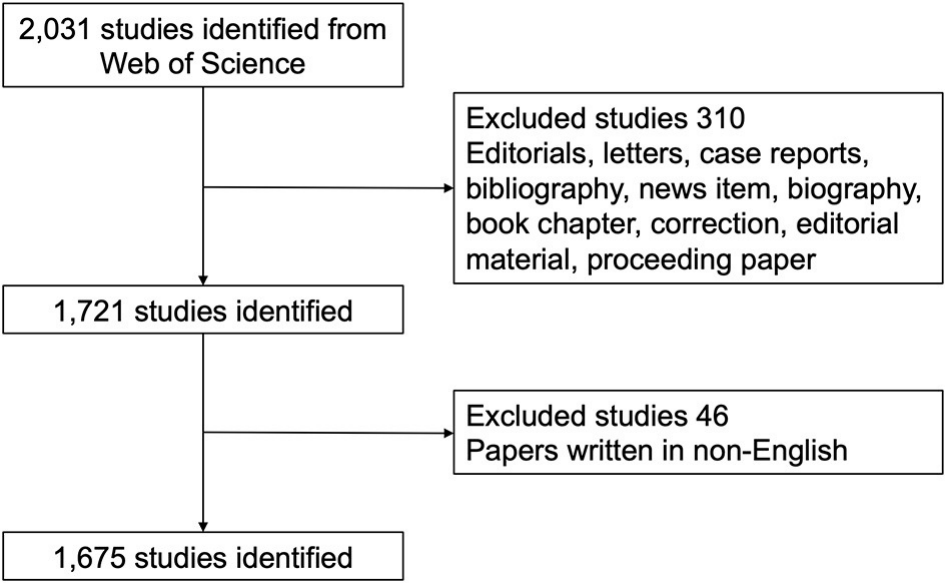
Flow diagram of the inclusion process. The detailed process of searching and screening.

All searches were performed on a single day, February 4, 2021, to avoid biases introduced by daily database renewal. The search strategies were integrated as follows: TI= (retinoblastoma) AND TS= (eye OR ocular OR oculus OR optical OR ophthalmic OR ophthalmology OR intraocular OR optic OR retinal OR retina) AND Language = English. Despite various types of manuscripts, only the original articles and reviews in the core database were included. Detailed procedures of the enrolment and screening were illustrated in Figure 1.

### Data Collection

The data were extracted carefully from all eligible publications, including titles, keywords, publication dates, countries and regions, institutions, authors, publishing journals, sums of citations, and H-indexes. Subsequently, the data were imported into Microsoft Excel 2010 (Redmond, Washington, USA) and VOSviewer (Leiden University, Leiden, the Netherlands) and analyzed both quantitatively and qualitatively.

### Bibliometric Analysis

#### Contribution of Countries to Global Publications

WOS was used to describe and examine the characteristics of all included publications. Microsoft Excel 2010 was applied to assess and rank the number of publications among different countries. To determine the global attention to the field, Relative research interest (RRI), which was defined as the number of publications in a specific field divided by all publications across all fields per year, was calculated.

#### Citations and H-Index

The information related to citations was acquired from the WOS database. The H-index means a scholar, a country or an author published H papers that have been cited in other publications at least H times, considered widely to reflect the scientific research impact of a scholar, a country, or an author (23).

#### Growth Trends of Publications

To predict the growth trends of publications in the field, Microsoft Excel 2010 was applied to generate the prediction model f(x) = ax 3 +bx 2+cx+d to calculate cumulative publications, by which we predicted future publication trends. In this formula, x represents time (year) and f (x) denotes the cumulative volume of publications in a certain year (24).

#### Journals, Institutions, and Authors Publishing Research

The top journals, institutions and authors and their number of publications were retrieved from WOS and Microsoft Excel 2010 was employed to illustrate the results.

#### Analysis of Keywords

VOSviewer was employed to map and visualize the network of keywords related to retinoblastoma research. Keywords were classified into disparate clusters according to co-occurrence analysis and simultaneously color-coded by time course. Furthermore, the average appearing year (AAY) was defined to estimate the novelty of a keyword (25).

## Results

### Assessment of Countries Contributing to Global Publications

**Figure 2:**
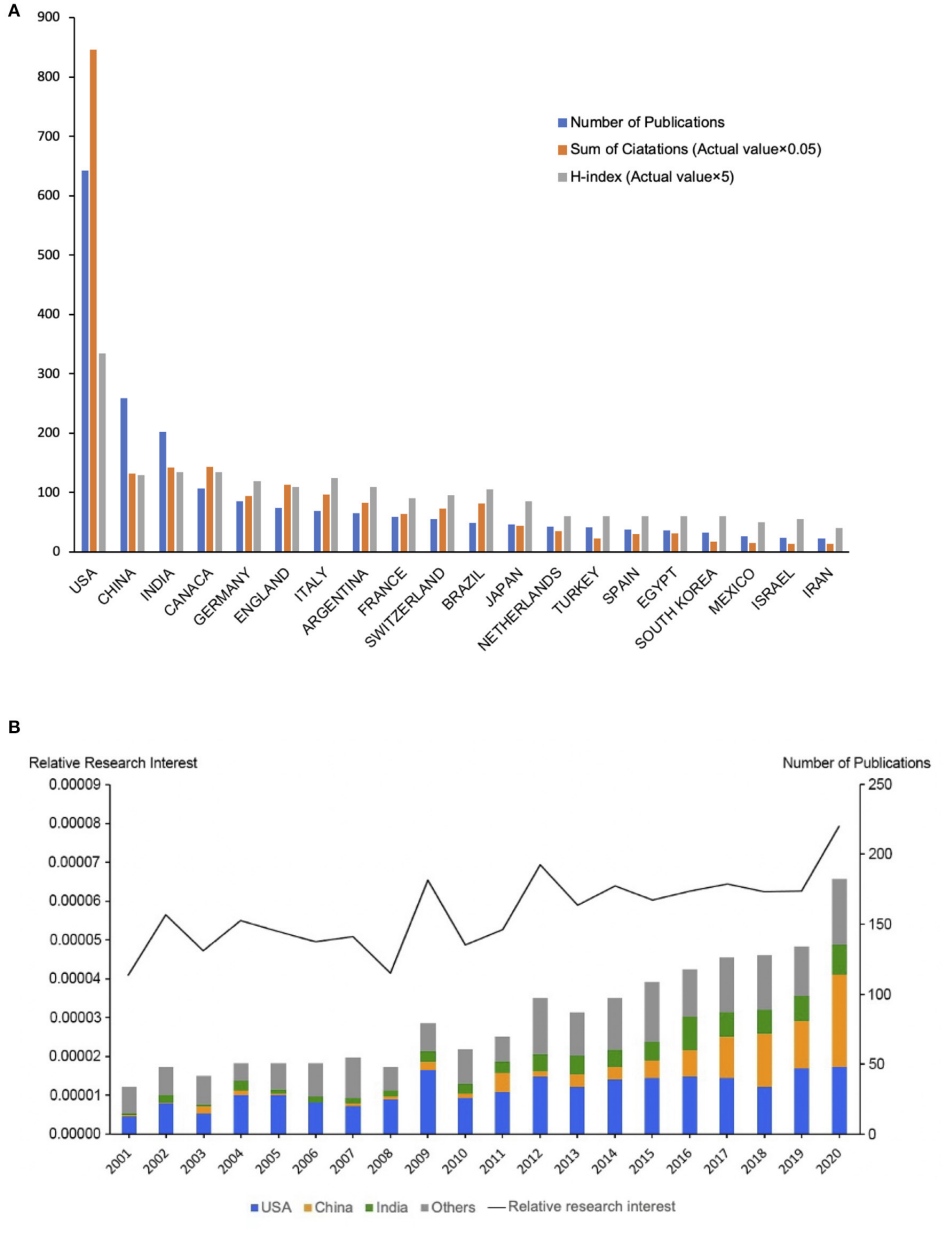
Contributions of different countries/regions to retinoblastoma research. **(A)** The number of publications, sum of citations (×0.05), and H-index (×5) of the top 20 countries or regions; **(B)** The number of publications worldwide and the top 3 countries per year are shown in the histogram. The time course of relative research interest is shown in the line chart.

A total of 1,675 articles from 2001 to 2021 met the search criteria. The United States ranked first in the number of publications (643, 38.4%), followed by China (259, 15.5%) and India (202, 12.1%) (Figure 2A). In relation to the number of publications per year, the year with the most publications was 2020 (182, 10.9%). Taking all-field publications into consideration, the global attention to this field based on the RRI value was ~0.005% before 2012, maintaining 0.006% from 2013 to 2019, and rising to 0.008% in 2019 (Figure 2B). While before 2011, China published no more than 6 papers in this field per year, the proportion of Chinese publications has increased rapidly over the last decade. Of note, China (66, 36.3%) exceeded the United States (48, 26.4%) in the number of annual publications each year for the first time in 2020.

### Citations and H-Index Analysis

Based on the citation report retrieved from the WOS database, all publications related to retinoblastoma have been cited 30,148 times since 2001 (15,469 citations without self-citations), with an average citing frequency of 18 times per paper. The most regularly cited papers were the papers from the United States, accounting for 56.2% of all citations (16,931 citations and 12,612 citations without self-citations) with an H-index of 67. Canada ranked second with a citation frequency of 2,864 (2,571 citations without self-citations) and an H-index of 27, followed by India with 2,829 citations (2,460 citations without self-citations) and an H-index of 27. Despite the second rank of China in the number of publications, the citation frequency ranked the fourth with 2,632 citations (2,230 citations without self-citations), and fourth with an H-index of 26 (Figure 2A).

### Growth Trends Prediction

**Figure 3:**
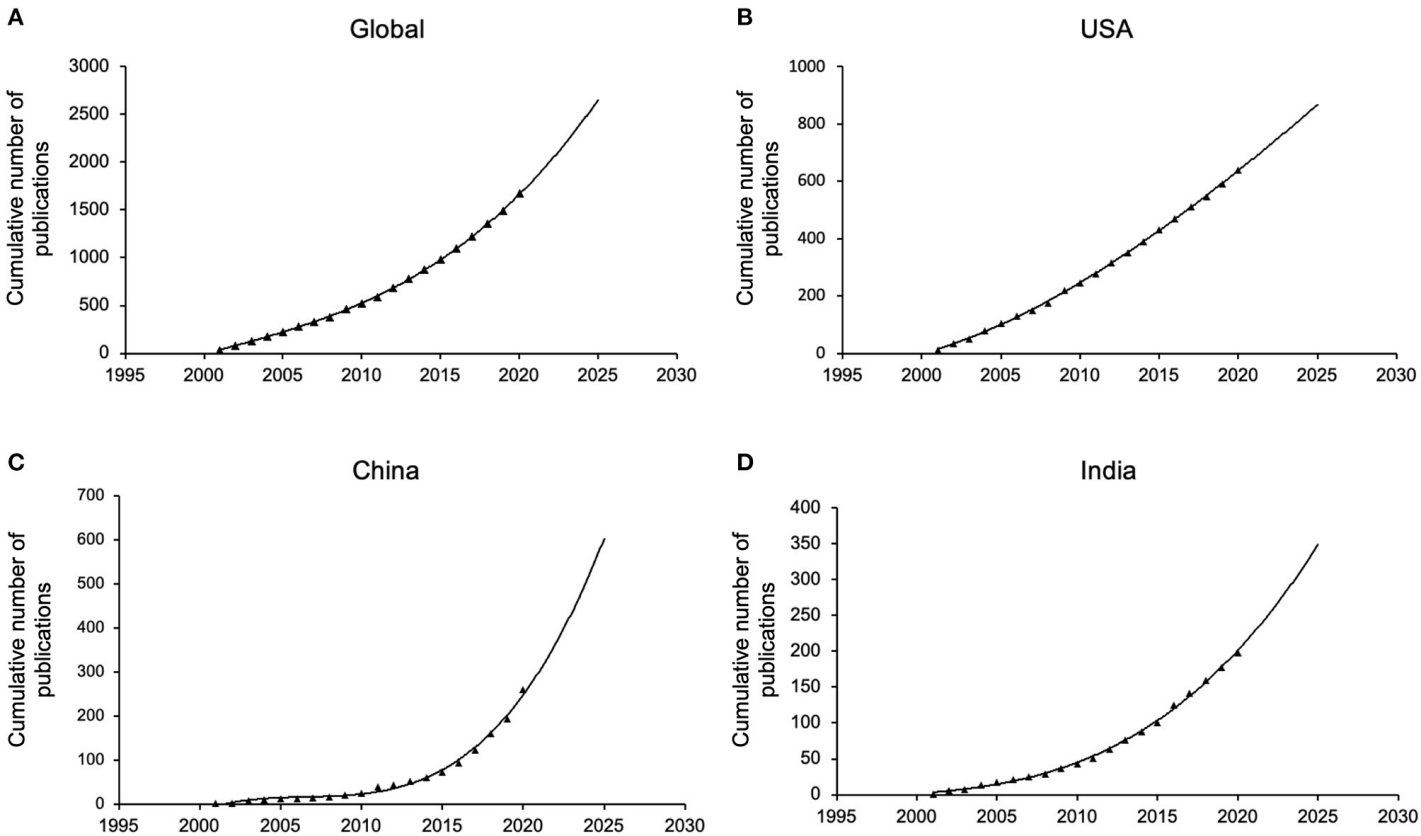
Fitting curves of publication growth trends concerning the retinoblastoma research. **(A)** Global; **(B)** the United States; **(C)** China; **(D)** India.

Model fitting curves of the growth in retinoblastoma publication demonstrated a significant correlation between time and a cumulative number of publications (Figure 3). Furthermore, publication trends for the following 5 years were estimated according to cumulative publication numbers over the past two decades. The volume of global publications increased at a steady and slow curve (Figure 3A), which is in accord with several major countries such as the United States and India (Figures 3B,D), while China has manifested an obviously faster growth (Figure 3C).

### Journals Publishing Research on Retinoblastoma

**Figure 4:**
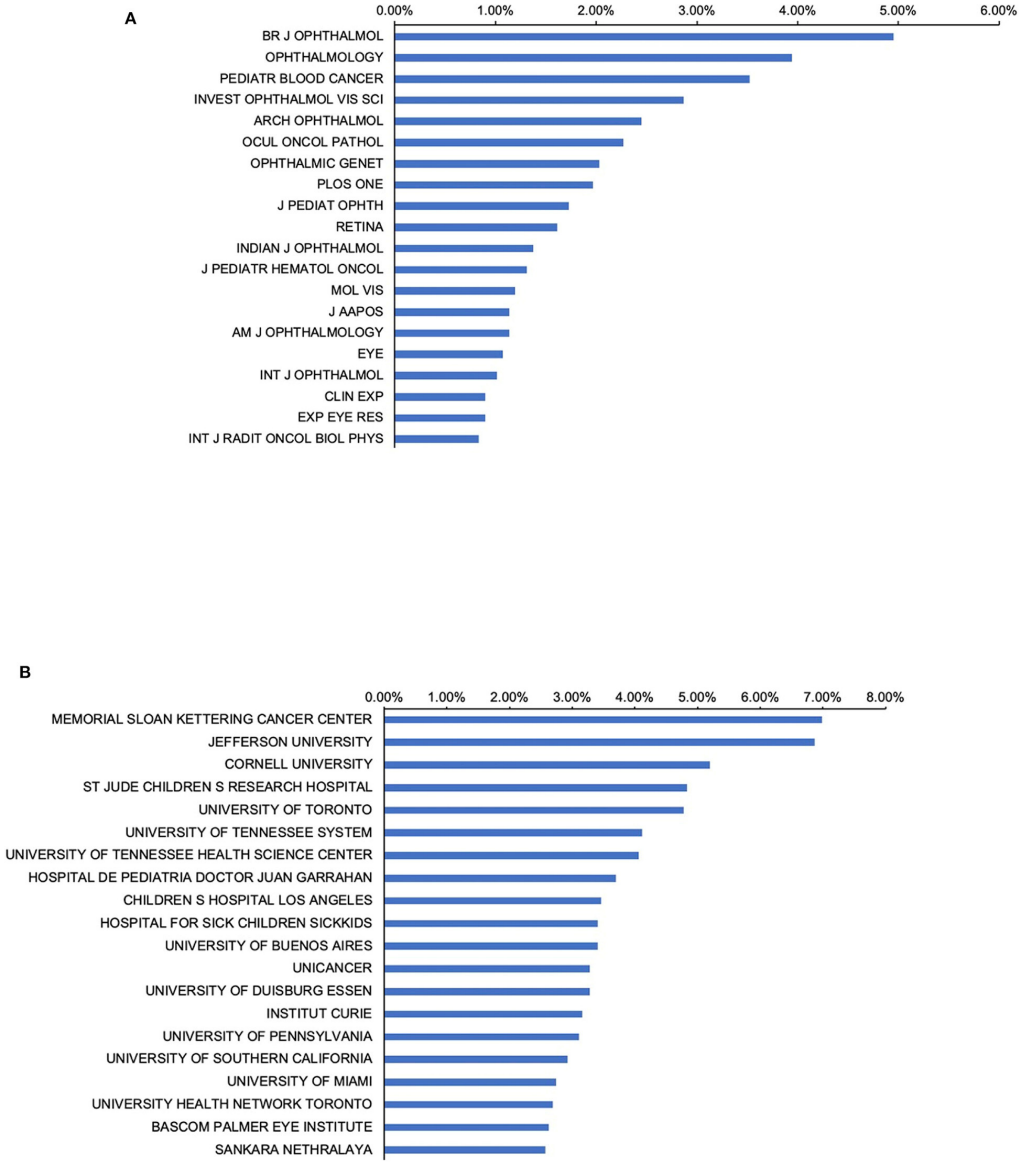
Distribution of journals and institutions focusing on retinoblastoma. **(A)** Distribution of the top 20 journals publishing research in the field; **(B)** Distribution of the top 20 institutes publishing research in the field.

More than one-third of the papers within this field were published in the 20 journals listed in Figure 4A (826, 38.7%), the *British Journal of Ophthalmology* published the most with 83 papers (5.0%), followed by *Ophthalmology* with 68 papers (3.9%). In addition, the number of papers published in the journal *Pediatric Blood Cancer* and *Investigative Ophthalmology Visual Science* was 66 (3.5%) and 59 (2.9%) records, ranking the third and the fourth, respectively. Other journals with high impacts, such as *Nature*, published three pieces of high-quality research in the related field, *Cell* published two and *Lancet* published one.

### Institutions Publishing Research on Retinoblastoma

Publications from the top 20 institutes accounted for 77.2% of all literature on retinoblastoma research (Figure 4B). Memorial Sloan Kettering Cancer Center in the United States had the highest number of publications (117, 7.0%) followed by Jefferson University in the United States (115, 6.9%). Within the top 20 institutions identified in the field, eleven are in the United States, three are Canadian institutions, two are in Argentina, two are in France, and one is in India.

### Authors Publishing Research on Retinoblastoma

A total of 598 papers were published by the top 10 authors, accounting for 35.7% of all literature in the field. Abramson DH of Memorial Sloan Kettering Cancer Center had published 114 papers related to retinoblastoma, ranking first in the number of publications (Table 1). Shields CL of Jefferson University ranked second with 103 publications; however, she had the highest H-index of 36. Shields JA of Jefferson University published 59 papers, ranking third. Among the top 10 authors, seven are from the United States, and the remaining three are from Argentina, Canada, and India, respectively.

**Table 1:** Top 10 authors with the most publications in retinoblastoma research.

**Author**	**Country**	**Affiliation**	**No. of publications**	**No. of citations**
ABRAMSON DH	USA	Memorial Sloan Kettering Cancer Center	114	3,768
SHIELDS CL	USA	Jefferson University	103	3,738
SHIELDS JA	USA	Jefferson University	59	2,949
DUNKEL IJ	USA	Memorial Sloan Kettering Cancer Center	56	2,070
FRANCIS JH	USA	Memorial Sloan Kettering Cancer Center	49	655
GALLIE BL	Canada	University of Toronto	47	1,826
CHANTADA GL	Argentina	Hospital De Pediatria Doctor Juan Garrahan	43	1,354
KRISHNAKUMAR S	India	Sankara Nethralaya	43	589
RODRIGUEZ-GALINDO C	USA	St Jude Children S Research Hospital	42	1,827
WILSON MW	USA	University of Tennessee Health Science Center	42	1,171

### Analysis of Keywords in Retinoblastoma Publications

**Figure 5:**
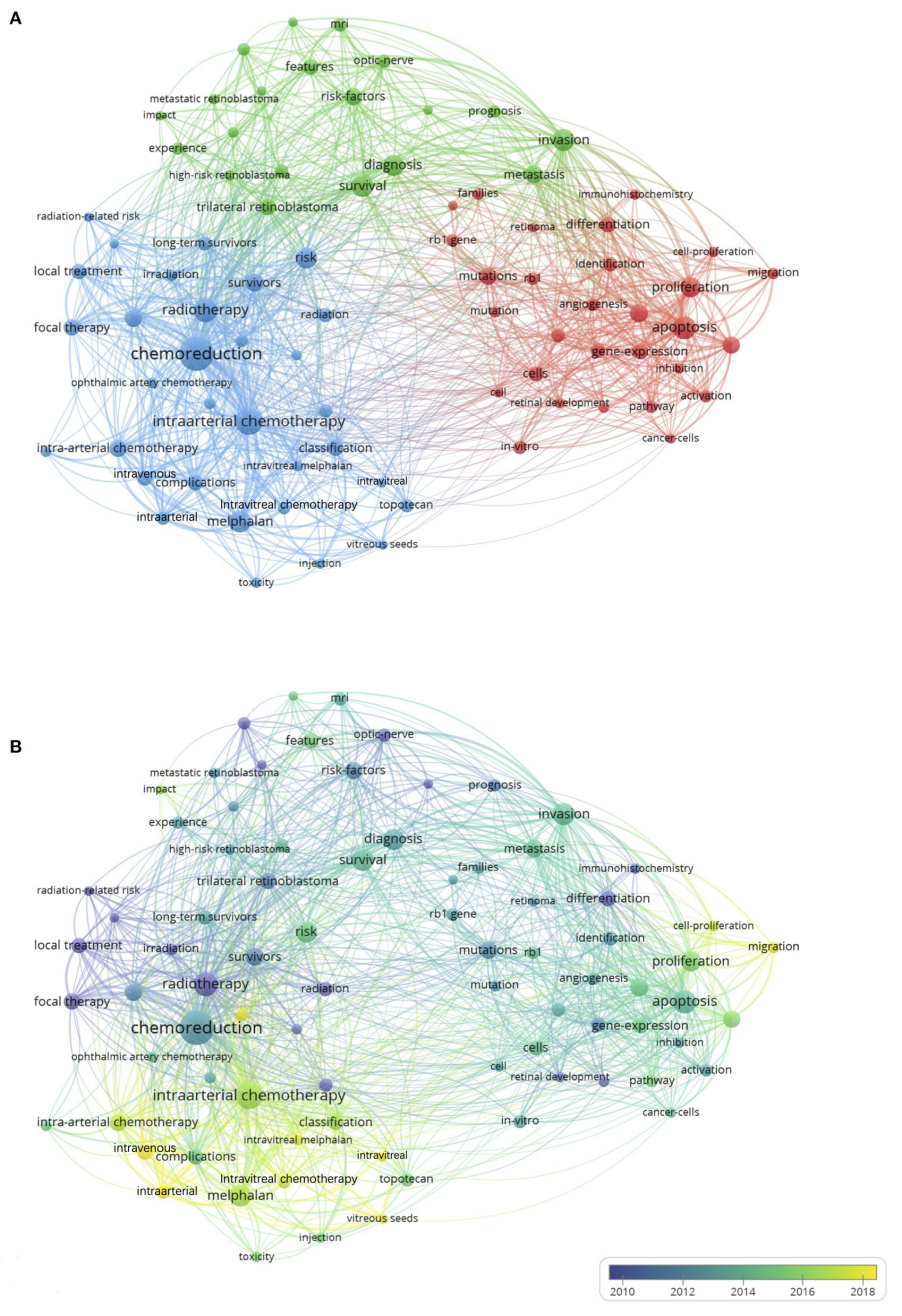
Analysis of keywords in publications of retinoblastoma. **(A)** Mapping of the keywords in the field. All keywords were divided into 3 clusters and colored differently: tumor-related research (left in green), clinical research (right in red), and management-related research (up in purple). The circle with a larger size indicates a higher frequency of the keyword; **(B)** Distribution of keywords on the basis of the average appearance time. Yellow represents a recent appearance, and blue represents an early appearance. The line between two keywords represents their co-occurrence in the same publication, with a thicker line indicating a closer relationship.

Keywords that occurred more than 25 times in the titles and abstracts extracted from 1675 publications were analyzed by using VOSviewer. After merging words with the same meaning words and excluding meaningless words, 81 keywords were identified and classified into tumor-related research, clinical research, and management-related research clusters (Figure 5A). Within the tumor-related research cluster, mentioned keywords were as follows: apoptosis (106 times), proliferation (87 times), and progression (72 times). With regard to the clinical research cluster, invasion (100 times), survival (103 times), and diagnosis (92 times) were the primary keywords. As with the management-related research cluster, the most common keywords comprised chemoreduction (316 times), intraarterial chemotherapy (309 times), and radiotherapy (123 times).

In addition, keywords were subsequently colored according to the AAY subsequently by VOSviewer (Figure 5B). Keywords that appeared relatively earlier were in blue, and keywords with a more recent appearance were in yellow. Keywords such as intravitreal (cluster 3, AAY of 2018.1), intraarterial (cluster 3, AAY of 2017.7), and intravenous (cluster 3, AAY of 2017.1) have emerged recently, while radiotherapy (cluster 3, AAY of 2010.0) was the major topic during the early stage. In addition, migration (cluster 1, AAY of 2017.6) and cell-proliferation (cluster 1, AAY of 2017.2) were the two newest words in the tumor-related research cluster. Of note, the most newly appearing words in the management-related research cluster indicate its trend in future investigation.

## Discussion

### Trends in Retinoblastoma Research

As is documented, the United States ranked first in terms of the total number of citations and H-index values in the field of retinoblastoma research, and undoubtedly, the United States contributed the most. Although retinoblastoma was first described as a distinct clinical entity by a Scottish surgeon in 1809, American scholars dedicated researching the disease earlier than researchers in the rest of the countries (11). In addition, the two-hit hypothesis was initially proposed by Alfred Knudson, an American physician and cancer geneticist, in 1971 (26), which shifted the concept of the cause of cancer from oncogene activation to tumor-suppressor loss of function, indicating that American scholars took the lead in comprehensively understanding the pathogenesis of retinoblastoma and developing early genetic testing and screening programmes. Moreover, the superior condition of both basic and clinical research in the United States provided adequate funding, advanced equipment, standardized systems and professional researchers. The fact that India ranked second in the sum of citations and H-index values may result from the largest burden of retinoblastoma carried by India, with almost 1,500 new cases every year (27).

Notably, China ranked second in the total number of publications; however, it ranked fourth in the sum of citations and H-index values, suggesting that the quality of research in the field of retinoblastoma still needs to be improved. The contradiction between the quantity and quality may be attributed to the lack of a standardized academic evaluation system, uneven competencies in clinical and scientific research among multiple institutions and the deficiency of high-quality multicenter randomized clinical trials (RCTs).

As illustrated in the time curve, steady growth in the cumulative number of publications concerning global retinoblastoma research was observed over time. In contrast to the United States, which maintained the annual publication quantity within a stable range as the global trend, China sustained a rapid development in the number of publications per year, which may be attributed to improved research conditions, increasingly dense academic networks and growing attention given to the disease over the last decade. Significantly, China accounted for one-third of the global number of publications in 2020, indicating the gradually indispensable position of China in the field.

Within the top 20 institutions in research regarding retinoblastoma, 11 institutions were from the United States, demonstrating its dominant status in the field. The fact that the United States occupies the most productive institutions across the world may partially explain why the United States consistently maintains its high quantity and quality of publications. Although China ranked next to the United States in the total publication quantity, none of the institutions in China was in the top 20, indicating the lack of institutions with professional and research stature with regard to retinoblastoma in China.

Remarkably, journals in the field of ophthalmology such as the *British Journal of Ophthalmology, Ophthalmology*, and *Investigative Ophthalmology Visual Science* were the primary journals involved in the publication of research on retinoblastoma. Therefore, it is reasonably concluded that future developments in the field are more likely to be published in these journals.

In relation to authors, Abramson DH of Memorial Sloan Kettering Cancer Center, Shields CL, and Shields JA of Jefferson University had published the most papers on retinoblastoma research. Abramson DH mainly evaluated and developed the management of retinoblastoma (28–30), and similarly, Shields CL focused on the investigation of optimizing the treatment for retinoblastoma patients but paid more attention to the establishment of an international classification to predict chemoreduction success (12). These scholars were considered leaders within the scope of retinoblastoma and their studies will continue to influence future research development and guide the cutting edge research on retinoblastoma.

### Focus in Retinoblastoma Research

Published papers that are cited frequently possess tremendous academic impact. The 10 publications with the highest citation frequency in retinoblastoma research are listed in Table 2. A paper entitled “Inactivation of the p53 pathway in retinoblastoma” published in *Nature* has been cited 427 times and is the most frequently cited paper in the field. The paper discovered the inactivation of the p53 pathway in retinoblastoma and revealed that the origin of retinoblastoma does not originate from intrinsically death-resistant cells, as previously thought (31). This perspective was in contrast to the prevailing theory proposed in *Cancer Cell* that retinoblastoma arises from extendedly proliferative cells with an intrinsically death-resistant capacity, which was cited 200 times as the seventh papers on the list (32). The second most highest cited paper, also published in *Nature* in 2012, supposed that the epigenetic deregulation of key cancer pathways caused by *RB1* loss may lead to the rapid development of retinoblastoma (33). In addition, a review entitled “retinoblastoma” and published in *Lancet* in 2012 presented the lessons learned about the management of retinoblastoma and proposed straightforward approaches to improve the survival chances and quality of life of children with retinoblastoma, which obtained the third most cited ranking (1).

**Table 2:** Top 10 publications with the most citations in retinoblastoma research.

**Title**	**Corresponding authors**	**Journal**	**Publication year**	**Total citations**
Inactivation of the p53 pathway in retinoblastoma	Michael A. Dyer	Nature	2006	427
A novel retinoblastoma therapy from genomic and epigenetic analyses	Michael A. Dyer James R. Downing Richard K. Wilson	Nature	2012	289
Retinoblastoma	Gallie, BL	Lancet	2012	288
The international classification of retinoblastoma predicts chemoreduction success	Shields, CL	Ophthalmology	2006	279
A phase I/II study of direct intraarterial (ophthalmic artery) chemotherapy with melphalan for intraocular retinoblastoma - Initial results	Abramson, DH	Ophthalmology	2008	259
Intra-arterial Chemotherapy for the Management of Retinoblastoma Four-Year Experience	Gobin, YP	Archives of Ophthalmology	2011	239
Cell-specific effects of RB or RB/p107 loss on retinal development implicate an intrinsically death-resistant cell-of-origin in retinoblastoma	Bremner, R	Cancer Cell	2004	200
Lifetime risks of common cancers among retinoblastoma survivors	Peto, J	Jnci-Journal of the National Cancer Institute	2004	174
Intravitreal chemotherapy for vitreous disease in retinoblastoma revisited: from prohibition to conditional indications	Munier, FL	British Journal of Ophthalmology	2012	173
Retinoblastoma management: advances in enucleation, intravenous chemoreduction, and intra-arterial chemotherapy	Shields, CL	Current Opinion in Ophthalmology	2010	169

Respecting the latest hotspot, intravitreal, intraarterial and intravenous chemotherapy from the management-related research cluster has emerged the most recently. As shown in Figures 5A,B, the cluster of management-related research achieved more attention than the other two clusters, suggesting that the management of retinoblastoma was continuously and extensively explored. With the emergence of IAC and IVitC, the indications for IVC are confined to patients with bilateral disease, confirmed germline mutation, family history or suspected optic nerve or choroidal invasion; IVC is also used as “bridge therapy” for patients weighing <6 kg awaiting IAC (34). Researchers have identified that IAC presented superior globe salvage in contrast to IVC for unilateral retinoblastoma patients in groups B, C, and D (11, 15, 35). Notably, IAC leads to 70% 5-year ocular survival for eyes with advanced retinoblastoma (36). Moreover, it has been reported that IAC is also feasible with low toxicity (37). IVitC is currently applied for refractory or recurrent vitreous seeds succeeding IAC or IVC (38, 39). A study revealed that intravitreal melphalan achieved 69.2% effectiveness in eyes with vitreous disease (40). A subsequent study demonstrated IVitC as a promising method for the treatment of vitreous seeds (41). Recently, it has been reported that intravitreal chemotherapy combined with endoresection seems to be safe and effective in globe-salvaging for eyes with refractory group D (42). In addition, with the increasing attention having been paid to the clinical use of anti-VEGF (vascular endothelial growth factor) agents in the patients with eye disease (43), a retrospective review suggested that intravitreal anti-VEGF contributed to a globe salvage rate of 51%, indicating the potential of intravitreal anti-VEGF in the conservative treatment of retinoblastoma (44). Despite a few studies on multiple types of chemotherapy, IVitC, IAC, and IVC are still focused on the field of retinoblastoma research, and optimal indications are explored to improve the global salvage and quality of life of retinoblastoma patients.

Recent years have witnessed a research focus on migration and cell-proliferation have become research focus in the field, indicating that the mechanisms concerning the development of retinoblastoma by regulating the migration and proliferation of retinoblastoma cells are still worth exploring. Emerging evidence has suggested a critical role of non-coding RNAs in the pathogenesis and progression of retinoblastoma. LncRNA SNHG14 was reported to be function as a competing endogenous RNA (ceRNA) of miR-124, upregulating signal transducer and activator of transcription 3 (STAT3); consequently, SNHG14 silencing inhibited cell proliferation, migration and invasion as well as promoted apoptosis in retinoblastoma cells (45). Similarly, the knockdown of LINC00324 decreased retinoblastoma cell proliferation, colony formation, migration, and invasion, and promoted apoptosis and cell cycle arrest with respect to the mechanism by which LINC00324 acted as a ceRNA for miR-769-5p which directly targeted STAT3 (46). Furthermore, circular RNAs (circRNAs) can also serve as ceRNAs to regulate the proliferation and migration of retinoblastoma cells, such as the circDHDDS/miR-361-3p/WNT3A axis and Circ_0000034/miR-361-3p/ADAM19 axis (47, 48). In addition to non-coding RNAs, cyclin-dependent kinase regulatory subunit 1B (CKS1B) downregulation hinders the proliferation, migration, and angiogenesis of retinoblastoma cells through the MEK/ ERK signaling pathway, and an anti-oncogene scavenger receptor class A member 5 (SCARA5) was reported to prevent the proliferation and migration of retinoblastoma cell lines by suppressing the PI3K/AKT signaling pathway (49, 50). Studies revealing the mechanisms of the pathogenesis and evolution of retinoblastoma will provide a promising theoretical basis for clinical therapy.

### Strengths and Limitations

Publications on retinoblastoma extracted from the WOS database were evaluated and analyzed comprehensively and objectively. However, there are still some inevitable limitations. For example, only publications in English were enrolled in the study; therefore, it is unavoidable that some important research in languages other than English were omitted. Moreover, the latest publications were not incorporated, which may in part affect our conclusions since they lack enough time to have accumulated considerable citations. Future investigations performing more complete search strategies and involving the latest and non-English language studies are expected. Of note, it is possible to mislabel document types by the literature database. As is reported that the WOS database has more improved accuracy in document type assignment than Scopus, we employed the WOS database to minimize the influence of mislabeling (51).

## Conclusions

The study has demonstrated global trends in retinoblastoma research. The United States has been at the cutting edge of the field based on its role as the lead contributor. Despite the considerable number of publications in China, the quality of the publications requires further improvement. Novel progress can be uncovered in the *British Journal of Ophthalmology* and *Ophthalmology*. Abramson DH and Shields CL are regarded as excellent candidates for academic collaboration in the field. Chemotherapy-related research has received the most attention previously and currently; furthermore, it may still be considered in the near future as the latest hotspot.

## Data Availability Statement

The original contributions presented in the study are included in the article/supplementary material, further inquiries can be directed to the corresponding author/s.

## Author Contributions

RJ and SG provided direction and guidance throughout the preparation of this manuscript. XG collected and interpreted the studies and was a major contributor to the writing and editing of the manuscript. MX reviewed and made significant revisions to the manuscript. All authors read and approved the final manuscript.

## Conflict of Interest

The authors declare that the research was conducted in the absence of any commercial or financial relationships that could be construed as a potential conflict of interest.

## Abbreviations

AAY: Average appearing year
CeRNA: Competing endogenous RNA
CircRNAs: Circular RNAs
CKS1B: Cyclin-dependent kinase regulatory subunit 1B
EBRT: External beam radiotherapy
IAC: Intraarterial chemotherapy
IVC: Intravenous chemotherapy
IVitC: Intravitreal chemotherapy
MRI: Magnetic resonance imaging
RRI: Relative research interest
SCARA5: Scavenger receptor class A member 5
SCI-E: Science Citation Index-Expanded
STAT3: Signal transducer and activator of transcription 3
VEGF: Vascular endothelial growth factor
WOS: Web of Science.
